# A Note on Beef-Tea

**Published:** 1904-06-11

**Authors:** Sydney Phillips

**Affiliations:** Steward of St. Thomas's Hospital.


### A NOTE ON BEEF-TEA.

By SYDNEY PHILLIPS, Steward of St. Thomas's Hospital.

It is reported that some of the London hospitals are abandoning the practice of making their own beef-tea and are purchasing instead certain meat extracts under the impression that they are effecting economy, and that they are getting at the same time a better article. As we have recently been importuned by pushing commercial travellers to use a well-known meat extract as a substitute for the beef-tea which is made in our hospital kitchen, it was decided to investigate the matter thoroughly and the results seem sufficiently interesting to make public. A surprise visit was paid to the hospital kitchen one morning and a quart sample of the beef-tea was taken and put into a bottle labelled A. Then the cook made up, in accordance with the printed instructions, a quart sample of beef-tea from the special meat extract. This was labelled B. The two samples were then sent to a chemist for examination. His analysis was as follows:—

| | A Hospital beef tea | B Special meat extract |
|---|---|---|
| Water ... ... ... ... ... | 96·030 | 97·096 |
| Fat ... ... ... ... ... | 0·199 | 0·029 |
| Insoluble proteids and meat fibre ... ... ... ... | 0·208 | 0·227 |
| Soluble proteids and gelatine | 1·342 | 0·652 |
| Meat bases ... ... ... ... | 0·608 | 0·761 |
| Non-nitrogenous extractive matters ... ... ... | 0·843 | 0·568 |
| Mineral matter ... ... ... | 0·770 | 0·667 |
| | 100·000 | 100·000 |
| Total dry solid matter ... | 3·970% | 2·904% |

The chemist proceeds to remark that "in criticising the above figures with a view to pronouncing an opinion as to the comparative nutritive value of these samples, it must be borne in mind that such preparations have extremely small, if any, value as real foods. They must be regarded as stimulants and restoratives, and this only in a limited sense, owing to the large proportion of water they contain. It will be noticed that the total dry solid matter amounts to only 3·970 per cent. in A and 2·904 per cent. in B. Deducting from these amounts respectively the sum of the meat bases, extractives and salts (valuable merely as stimulants), together with the proportion of fat and also disregarding the vexed question as to the real nutritive power of gelatine, there is left to A 1·55 per cent. nitrogenous organic matter, and to B 0·879 per cent., and these figures therefore become a fair index of their relative nutritive value." That is to say, our own beef tea was the more nutritious.

An examination of the cost of the two articles further showed that the meat extract was somewhat dearer than our own preparation.

The result is one that perhaps might have been anticipated. It seems scarcely reasonable that a company, which has to spend large sums on advertisements, directors' fees, and travellers' commissions, besides providing dividends for its shareholders, could produce a cheaper or better article than a hospital where practically the only expense is the purchase of the raw material.

